# Analysis of risk factors and short-term prognostic factors of arrhythmia in patients infected with mild/moderate SARS-CoV-2 Omicron variant

**DOI:** 10.3389/fmed.2023.1186200

**Published:** 2023-07-27

**Authors:** Lijie Yan, Jintao Wu, Xianwei Fan, Jingjing Liu, Leiming Zhang, Juan Hu, Xuejie Li, Yandong Su, Futao Zhang, Xizheng Xu, Xiaosheng Chen, Haitao Yang

**Affiliations:** ^1^Heart Center of Henan Provincial People’s Hospital, Fuwai Central China Cardiovascular Hospital, Fuwai Central China Hospital of Zhengzhou University, Zhengzhou, Henan, China; ^2^Department of Cardiology, Henan University People’s Hospital, Henan Provincial People’s Hospital, Zhengzhou, Henan, China

**Keywords:** SARS-CoV-2, Omicron, arrhythmia, risk factors, prognosis

## Abstract

**Background:**

Complications, including arrhythmia, following severe acute respiratory syndrome-coronavirus-2 (SARS-CoV-2) infection continue to be of concern. Omicron is the mainstream SARS-CoV-2 mutant circulating in mainland China. At present, there are few epidemiological studies concerning the relationship between arrhythmia and Omicron variant infection in mainland China.

**Objectives:**

To investigate the risk factors of arrhythmia in patients infected with the SARS-CoV-2 Omicron variant and the factors influencing prognosis.

**Methods:**

Data from 192 Omicron infected patients with symptoms of arrhythmia (AH group) and 100 Omicron infected patients without arrhythmia (Control group) were collected. Patients in the AH group were divided into the good and poor prognosis groups, according to the follow-up results 4–6 weeks after infection. The general and clinical data between the AH and Control groups, and between the good and poor prognosis groups were compared. The variables with differences between the groups were included in the multivariate logistic regression analysis, and the quantitative variables were analyzed by receiver operating characteristic curve to obtain their cut-off values.

**Results:**

Compared with the control group, the body mass index (BMI), proportion of patients with a history of arrhythmia, proportion of antibiotics taken, heart rate, moderate disease severity, white blood cell (WBC) count, and the aspartate aminotransferase, creatine kinase (CK), CK isoenzyme (CK-MB), myoglobin (Mb), high-sensitive troponin I (hs-cTnI), lymphocyte ratio and high sensitivity C-reactive protein (hs-CRP) levels in the AH group were significantly higher (*p* < 0.05). In addition, obesity (BMI ≥24 kg/m^2^), fast heart rate (≥100 times/min), moderate disease severity, and WBC, CK-MB and hs-cTnI levels were independent risk factors of arrhythmia for patients with Omicron infection (*p* < 0.05), and hs-CRP was a protective factor (*p* < 0.05). Compared with the good prognosis group, the age, proportion of patients with a history of arrhythmia, heart rate, proportion of moderate disease severity, and hs-CRP, CK, Mb and hs-cTnI levels were significantly higher in the poor prognosis group, while the proportion of vaccination was lower in the poor prognosis group (*p* < 0.05). Advanced age (≥65 years old), proportion of history of arrhythmia, moderate disease severity, vaccination, and hs-CRP, Mb and cTnI levels were independent factors for poor prognosis of patients with arrhythmia (*p* < 0.05).

**Conclusion:**

The factors that affect arrhythmia and the prognosis of patients infected with Omicron include obesity, high heart rate, severity of the disease, age. history of arrhythmia, WBC, hs-CRP, and myocardial injury indexes, which could be used to evaluate and prevent arrhythmia complications in patients in the future.

## 1. Introduction

The coronavirus disease 2019 (COVID-19) global pandemic, caused by severe acute respiratory syndrome-coronavirus-2 (SARS-CoV-2), has had a huge impact on public health. The virus also mutates continuously due to sustained and widespread transmission (1). The Omicron variant emerged in late 2021 and is currently the most prevalent strain in mainland China (2). Myocardial damage, one of the main manifestations of which is arrhythmia, is a complication after infection with SARS-CoV-2 as it can directly invade the respiratory system and the heart. SARS-CoV-2 participates in the progression of myocardial injury and causes persistent arrhythmias by inducing mechanisms such as hypoxemia, myocardial local inflammation, physiological changes in myocardial ion channels, myocardial immune activation and autonomic nervous disorders (3, 4). A study has confirmed the close relationship between SARS-CoV-2 infection and atrial fibrillation and other arrhythmias, and it is the main cause of cardiovascular-related death in patients with COVID-19 (5).

Cardiac arrhythmia is a major determinant for the morbidity and mortality of patients with COVID-19 (6–8), especially in acute settings. Han et al. (9) demonstrated that SARS-CoV-2 could infect human sinoatrial node-like pacemaker cells in hamsters and cause arrhythmia. At present, few studies have focused on the relationship between Omicron variant infection and arrhythmia, particularly in mainland China. Existing studies, mostly conducted before the development of Omicron, have mainly involved other variants of SARS-CoV-2, such as the Delta variant, or have not distinguished the type of strain patients were infected with (10, 11). Many studies have reported that Omicron infection has milder symptoms compared with the previous variant strains, and it is believed that this is related to differences in the mechanism in which the virus enters the cell, the action of the vaccine and the virulence (12–14). Varshney and Agarwal (11) reported the incidence of arrhythmias in patients with COVID-19 with Delta B.1.617 and found that sinus bradycardia was the most common type of arrhythmia, with the second being atrial fibrillation. However, our clinical experience indicates that tachyarrhythmia is the most common type of arrhythmia in patients infected with Omicron. Therefore, it is believed that patients infected with Omicron have different clinical characteristics in relation to arrhythmias, compared with other variant strains.

Before December 2022, the prevalence of Omicron variants was largely limited in mainland China due to strict epidemic prevention and control policies. Therefore, studies on the correlation between Omicron variants and arrhythmia complications in mainland China were rarely conducted due to the lack of cases. Since the State Council of China issued “the Notice on Further Optimizing and Implementing the Prevention and Control Measures of the Novel Coronavirus” (also known as the “New Ten Measures”), the epidemic prevention policy has been gradually relaxed, and the number of patients infected with the Omicron variant has markedly increased. In the present study, the data of 192 Omicron-infected patients with arrhythmia symptoms were collected, and the short-term prognosis of the patients was followed up to explore the factors affecting the occurrence and prognosis of arrhythmia. To the best of our knowledge, this study is the first to investigate the epidemiological factors of arrhythmia in mild/moderate Omicron infection in mainland China.

## 2. Data and methods

### 2.1. Cases

This study was a prospective randomized controlled study of COVID-19 patients from the Henan Provincial People’s Hospital from December 2022 to February 2023. The inclusion criteria were as follows: (1) permanent resident of mainland China aged >18 years old; (2) the clinical manifestations were consistent with the diagnosis criteria of COVID-19, and the pathogen was confirmed as the Omicron variant by laboratory examination (PCR); (3) the clinical diagnosis was mild or moderate disease (15); (4) ECG examination confirmed the combination of tachycardia (symptomatic), atrial fibrillation, premature beat, atrioventricular block, or with arrhythmia symptoms for example, palpitations, chest tightness, dizziness, sweating, syncope, and Art-S syndrome and so on. The exclusion criteria were as follows: (1) incomplete medical history or follow-up data; (2) patients with cardiac pacemakers; (3) patients with hyperthyroidism; (4) patients with congenital heart disease; (5) patients with chronic kidney disease, immune system diseases, malignant tumors and other special diseases by chief complaint; (6) pregnant or lactating women. The eliminated criteria were as follows: (1) poor treatment compliance or withdrawal of treatment; (2) the disease progressed to severe, critical or death. This study intends to collect about 200 patients meeting the study conditions and define as AH group, and according to the principle of age and sex matching, control individuals (control group) were selected from Omicron infected patients not suffering from arrhythmia (confirmed by ECG). All patients provided informed consent to participate in the study, and this study was approved by the Ethics Committee of the Henan Provincial People’s Hospital.

### 2.2. Data

#### 2.2.1. General data

The age, sex, body mass index (BMI), smoking, history of hypertension, diabetes, cerebrovascular disease, heart disease, history of arrhythmia and vaccination status (including completely + booster vaccinated, completely vaccinated or partially vaccinated) data were collected for all patients, according to the patient electronic medical records and chief complaint data. Self-administration of antibiotics data were also collected. Some patients blindly took common antibiotics at the onset of symptoms, which is common in China. The administered antibiotics were limited to four types: Cefin, amoxicillin, roxithromycin and penicillin.

#### 2.2.2. Clinical symptoms

Maximum body temperature, heart rate (HR), respiratory rate, mean arterial pressure and pulse oxygen saturation of patients at the first visit were recorded. Disease severity [mild or moderate using the “Diagnosis and Treatment Protocol for Novel Coronavirus Infection (Trial 10th edition)” diagnostic criteria] was determined in combination with lung imaging examination (15).

#### 2.2.3. Laboratory indicators

A total of 5 mL fasting venous blood from each patient was collected and sent to the laboratory for a blood routine test and liver function, kidney function and myocardial enzyme profile tests. White blood cell (WBC) count, lymphocyte proportion (LYM%), high sensitivity C-reactive protein (hs-CRP), serum creatinine (Scr), blood urea nitrogen (BUN), alanine aminotransferase (ALT), aspartate aminotransferase (AST), lactate dehydrogenase (LDH), creatine kinase (CK), CK isoenzyme (CK-MB), myoglobin (Mb) and high-sensitive troponin I (hs-cTnI) were measured. The normal range of each index was defined as: WBC, 4–9 × 10^9^/L; LYM%, 20–40%; hs-CRP, 0-10 mg/L; Scr, 0–106 μmol/L for men and 0–97 μmol/L for women; BUN, 3.2–7.1 mmol/L; ALT, 5–40 U/L for men and 5–35 U/L for women; AST, 0–40 U/L; LDH, 0–240 U/L; CK, 38–174 U/L for men and 26–140 U/L for women; CK-MB, 0–5 ng/mL; Mb, 0–72 U/L for men and 0–58 U/L for women; hs-cTnI, 0–30 ng/L.

### 2.3. Treatment and follow-up

All patients were administered antiviral (Azvudine tablets) or symptomatic treatment according to the severity of illness and given support to improve myocardial energy metabolism according to the level of myocardial injury (to inhibit oxidative stress response after pneumonia) and hormone therapy if necessary. After 4–6 weeks of treatment, all patients were requested by telephone to present for follow-up to confirm whether their condition had improved according to their ECG findings. In the course of the study, it was found that most patients with arrhythmia significantly improved or recovered as the virus RNA detection became negative and the respiratory symptoms disappeared, but there are also some patients whose symptoms failed to improve or worsened in ≥6 weeks. Therefore, using the 6-week limit, the short-term prognosis of patients were defined as either recovery of COVID-19 (negative viral RNA test and respiratory symptoms had mostly disappeared), good prognosis (complete disappearance or significant remission of arrhythmia symptoms) or poor prognosis.

### 2.4. Statistical analysis

The general and clinical data between the AH group and the control group or the good prognosis group and the poor prognosis group were compared. The count data are presented as the number of cases and percentage, the comparison between groups was conducted using the Chi-square test, the measurement data in-line with the normal distribution were presented as the mean and standard deviation, and the independent sample unpaired *t-*test was used for the comparison between groups. Logistic regression was conducted to analyze the influencing factors of patients with arrhythmia and poor prognosis, and the measurement variables were converted into binary variables according to clinical diagnosis and the regression method was “forward-condition”. The receiver operating characteristic curve (ROC) was used to analyze the optimal cut-off value of the measurements. In the statistical process, *p* > 0.05 was considered to indicate a statistically significant difference.

## 3. Results

### 3.1. General description

**Figure 1:**
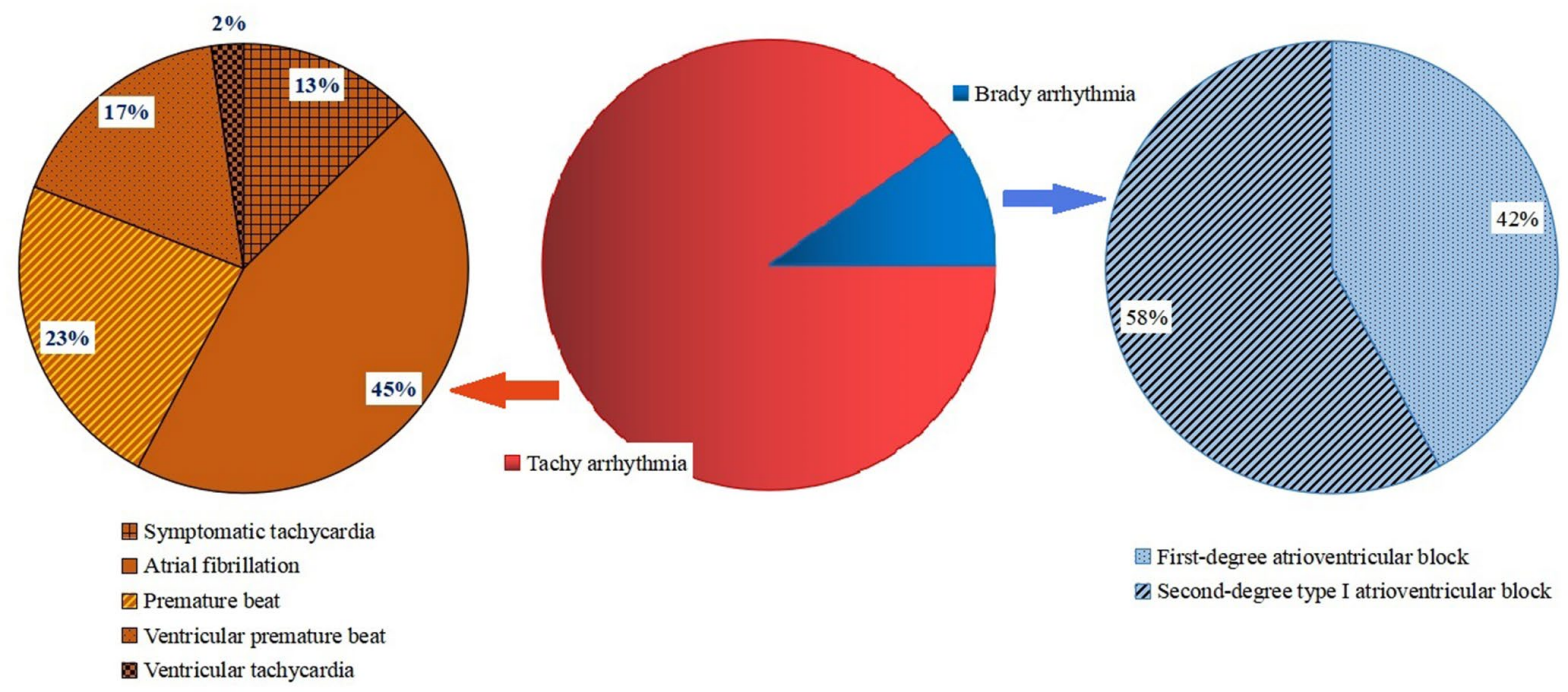
Distribution diagram of types of arrhythmias in patients infected with Omicron COVID-19.

Among the 192 Omicron-infected patients with arrhythmia, 173 cases (90.1%) had tachyarrhythmia, including 22 cases (11.5%) of symptomatic tachycardia, 78 cases (40.6%) of atrial fibrillation, 40 cases (20.8%) of premature beat, 29 cases (15.1%) of ventricular premature beat and 4 cases (2.1%) of ventricular tachycardia. A total of 19 cases (9.9%) had brady arrhythmia, including 8 cases (4.2%) of first-degree atrioventricular block and 11 cases (5.7%) of second-degree type I atrioventricular block. After 4–6 weeks of symptomatic treatment, 147 patients (76.6%) recovered and their arrhythmia symptoms mostly disappeared, while 45 patients (23.4%) had no obvious relief of arrhythmia symptoms, which was regarded as poor prognosis (Figure 1).

### 3.2. Comparison of data between the AH and control groups

The general and clinical data between the AH and the control groups were compared. The results indicated that the BMI, proportion of patients with arrhythmia history, proportion of antibiotic taken, HR, proportion of moderate disease severity and WBC, AST, CK, CK-MB, Mb and hs-cTnI levels in the AH group were significantly higher than those in the control group (*p* < 0.05), and the lymphocyte ratio and hs-CRP levels were significantly lower than those in the control group (Table 1; *p* < 0.05).

**Table 1:** Comparison of the data of the AH and control groups [(*x̅* ± s)/*n* (%)].

Data	AH group (*n* = 192)	Control group (*n* = 100)	*t*/*χ*^2^	*p*
Age (years)	49.85 ± 19.51	49.61 ± 16.32	0.113	0.910
Sex [*n* (%)]				
Male	97 (50.5)	42 (42.0)	1.914	0.167
Female	95 (49.5)	58 (58.0)		
BMI (kg/m^2^)	22.82 ± 2.7	21.07 ± 2.17	5.620	0.000
Smoke [*n* (%)]	52 (27.1)	28 (28.0)	0.028	0.868
Hypertension history [*n* (%)]	68 (35.41)	35 (35.0)	0.005	0.944
Diabetes history [*n* (%)]	22 (11.4)	12 (12.0)	0.019	0.891
Cerebrovascular disease history [*n* (%)]	25 (13.0)	7 (7.0)	2.443	0.118
Arrhythmia history [*n* (%)]	36 (18.8)	9 (9.0)	4.795	0.029
Cardiopathy history [*n* (%)]	25 (13.0)	7 (7.0)	2.443	0.118
Vaccination [*n* (%)]	164 (85.4)	88 (88.0)	0.371	0.542
Take antibiotics [*n* (%)]	61 (31.8)	16 (16.0)	8.423	0.004
Antiviral therapy [Azvudine, *n* (%)]	42 (21.4)	15 (15.0)	1.978	0.160
Body temperature (°)	38.72 ± 0.69	38.76 ± 0.7	−0.488	0.626
HR (times/min)	92.09 ± 15.84	85.27 ± 12.96	3.709	0.000
Respiratory rate (times/min)	20.03 ± 3.11	20.12 ± 3.01	−0.234	0.815
MAP (mm Hg)	93.24 ± 13.01	90.31 ± 11.71	1.892	0.060
SPO_2_ (%)	97.93 ± 1.63	97.72 ± 1.47	1.089	0.277
Severity [*n* (%)]				
Mild	164 (85.4)	97 (97.0)	9.297	0.002
Moderate	28 (14.6)	3 (3.0)		
WBC (10^9/L)	8.66 ± 3.88	6.36 ± 2.92	5.675	0.000
LYM% (%)	36.69 ± 7.28	38.52 ± 7.42	−2.022	0.044
hs-CRP (mg/L)	7.93 ± 3.47	8.78 ± 3.4	−1.997	0.047
Scr (μmol/L)	86.28 ± 27.99	92.44 ± 29.35	−1.756	0.080
BUN (mmol/L)	5.06 ± 1.37	5.14 ± 1.34	−0.447	0.655
AST (U/L)	37.69 ± 36.57	28.27 ± 28.69	2.241	0.026
ALT (U/L)	37.7 ± 35.49	30.53 ± 29.32	1.841	0.067
LDH (U/L)	238.69 ± 58.25	227.38 ± 45.32	1.83	0.068
CK (U/L)	121.57 ± 44.89	73.99 ± 35.21	9.945	0.000
CK-Mb (ng/mL)	5.17 ± 1.8	1.06 ± 0.97	25.309	0.000
Mb (U/L)	72.48 ± 50.52	29.17 ± 15.58	10.924	0.000
hs-cTnI (ng/L)	105.52 ± 137.06	12.03 ± 8.15	9.42	0.000

HR, heart rate; MAP, mean arterial pressure; SPO_2_, pulse oxygen saturation; WBC, white blood cell (count); LYM%, lymphocyte percentage; hs-CRP, high sensitivity C-reactive protein; Scr, serum creatinine; BUN, blood urea nitrogen; ALT, alanine aminotransferase; AST, aspartate aminotransferase; LDH, lactate dehydrogenase; CK, creatine kinase; CK-MB, creatine kinase isoenzyme; Mb, myoglobin; hs-cTnI, high-sensitive troponin I.

### 3.3. Analysis of influencing factors of complicated arrhythmia

To exclude the interaction between the factors, the variables with statistically significant differences (*p* < 0.05) between the AH and control groups were included in a multivariate logistic regression analysis. The results demonstrated that obesity (BMI ≥24 kg/m^2^), high HR (>100/min), moderate disease severity, increased CK-MB and hs-cTnI were independent risk factors for arrhythmia in infected patients (*p* < 0.05), while elevated hs-CRP was a protective factor (Table 2; *p* < 0.05).

**Table 2:** Multivariate analysis of factors in arrhythmia patients infected with Omicron COVID-19.

Variables	B	SE	Wals	*p*	OR	95% CI of OR	
						Lower	Upper
BMI2 (≥24 kg/m^2^)	1.355	0.447	9.179	0.002	3.877	1.613	9.314
HR (≥100 times/min)	1.165	0.406	8.242	0.004	3.205	1.447	7.097
Severity (Moderate)	2.673	0.753	12.589	0.000	14.486	3.309	63.425
WBC			23.800	0.000			
WBC (<4 × 10^9/L)	−0.912	0.495	3.399	0.065	0.402	0.152	1.059
WBC (>10 × 10^9/L)	1.881	0.455	17.132	0.000	6.563	2.693	15.995
hs-CRP (>10 mg/L)	−0.732	0.373	3.847	0.050	0.481	0.231	0.999
CK-Mb (>5 ng/mL)	3.104	0.558	30.998	0.000	22.287	7.473	66.468
CTnI (>30 ng/L)	2.398	0.495	23.416	0.000	10.998	4.164	29.044

### 3.4. Comparison of data between arrhythmia patients in the poor and good prognosis groups

The general and clinical data of AH patients in the poor prognosis and good prognosis groups were compared. The results demonstrated that, compared with the good prognosis group, age, proportion of patients with a history of arrhythmia, HR, proportion of moderate disease severity and CK, Mb and hs-cTnI levels in the poor prognosis group were significantly higher (Table 3; *p* < 0.05), and the vaccination proportion was significantly lower compared with the good prognosis group (*p* < 0.05).

**Table 3:** Comparison of the data of the good prognosis and poor prognosis groups [(*x̅* ± s)/*n* (%)].

Data	Good prognosis group (*n* = 147)	Poor prognosis group (*n* = 45)	*t*/*χ*^2^	*p*
Age (years)	46.57 ± 18.47	60.58 ± 19.13	−4.414	0.000
Sex [*n* (%)]				
Male	71(48.3)	24(53.3)	0.349	0.555
Female	76(51.7)	21(46.7)		
BMI (kg/m^2^)	22.61 ± 2.65	23.5 ± 2.77	−1.946	0.053
Smoke [*n* (%)]	41(27.9)	11(24.4)	0.207	0.649
Hypertension history [*n* (%)]	55(37.4)	13(28.9)	1.095	0.295
Diabetes history [*n* (%)]	15(10.2)	7(15.6)	0.973	0.324
Cerebrovascular disease history [*n* (%)]	22(15.0)	3(6.7)	2.095	0.148
Arrhythmia history [*n* (%)]	21(14.3)	15(33.3)	8.205	0.004
Cardiopathy history [*n* (%)]	16(10.9)	9(20.0)	2.528	0.112
Vaccination [*n* (%)]	134(91.2)	29(64.4)	19.172	0.000
Take antibiotics [*n* (%)]	45(30.6)	16(35.6)	0.388	0.533
Antiviral therapy [Azvudine, *n* (%)]	29(19.7)	13(28.9)	1.692	0.193
Body temperature (°)	38.72 ± 0.69	38.73 ± 0.67	−0.122	0.903
HR (times/min)	91.82 ± 15.77	92.98 ± 16.21	−0.427	0.670
Respiratory rate (times/min)	20.14 ± 3.03	19.67 ± 3.36	0.898	0.370
MAP (mm Hg)	93.73 ± 13.02	91.64 ± 12.97	0.943	0.347
SPO_2_ (%)	97.78 ± 1.26	97.33 ± 1.77	1.583	0.119
Severity [*n* (%)]				
Mild	134(91.2)	30(66.7)	6.889	0.009
Moderate	13(8.8)	15(33.3)		
WBC (10^9/L)	8.69 ± 3.97	8.57 ± 3.6	0.171	0.865
LYM% (%)	37 ± 7.35	35.69 ± 7.03	1.058	0.291
hs-CRP (mg/L)	7.54 ± 3.23	9.2 ± 3.94	−2.587	0.012
Scr (μmol/L)	85.11 ± 29.12	90.09 ± 23.83	1.045	0.298
BUN (mmol/L)	5.16 ± 1.36	4.73 ± 1.39	1.838	0.068
AST (U/L)	35.06 ± 30.66	45.91 ± 45.57	−1.497	0.140
ALT (U/L)	37.18 ± 36.41	39.4 ± 32.42	−0.367	0.714
LDH (U/L)	238.83 ± 56.26	238.24 ± 64.99	0.059	0.953
CK (U/L)	117.81 ± 42.42	133.84 ± 50.73	2.115	0.036
CKMB (ng/mL)	5.2 ± 1.84	5.06 ± 1.7	0.476	0.635
Mb (U/L)	62.59 ± 42	104.8 ± 61.88	4.283	0.000
hs-cTnI (ng/L)	91.75 ± 125.7	152.2 ± 161.62	2.631	0.009

### 3.5. Analysis of prognostic factors of arrhythmia

To exclude the interaction between the factors, the variables with statistically significant differences (*p* < 0.05) between the good prognosis and poor prognosis groups were also included in a multivariate logistic regression analysis. The results demonstrated that advanced age (≥65 years old), history of arrhythmia, moderate disease severity and higher hs-CRP, Mb and hs-cTnI levels were independent risk factors for poor prognosis in Omicron-infected patients suffering from arrhythmia (*p* < 0.05), while vaccination was a protective factor (Table 4; *p* < 0.05).

**Table 4:** Multivariate analysis of prognosis factors in arrhythmia patients infected with Omicron COVID-19.

Variables	B	SE	Wals	*p*	OR	95% CI of OR	
						Lower	Upper
Age (≥65 years)	1.367	0.447	9.342	0.002	3.925	1.633	9.433
Arrhythmia history	1.015	0.522	3.775	0.052	2.759	0.991	7.681
Vaccination	−1.225	0.516	5.634	0.018	0.294	0.107	0.808
Severity (Moderate)	1.844	0.534	11.901	0.001	6.319	2.217	18.011
hs-CRP (>10 mg/L)	1.542	0.491	9.872	0.002	4.675	1.786	12.234
Mb (Male<72 U/L or female <58 U/L)	2.000	0.478	17.539	0.000	7.389	2.898	18.838
cTnI (>30 ng/L)	0.956	0.457	4.373	0.037	2.601	1.062	6.371

**Figure 2:**
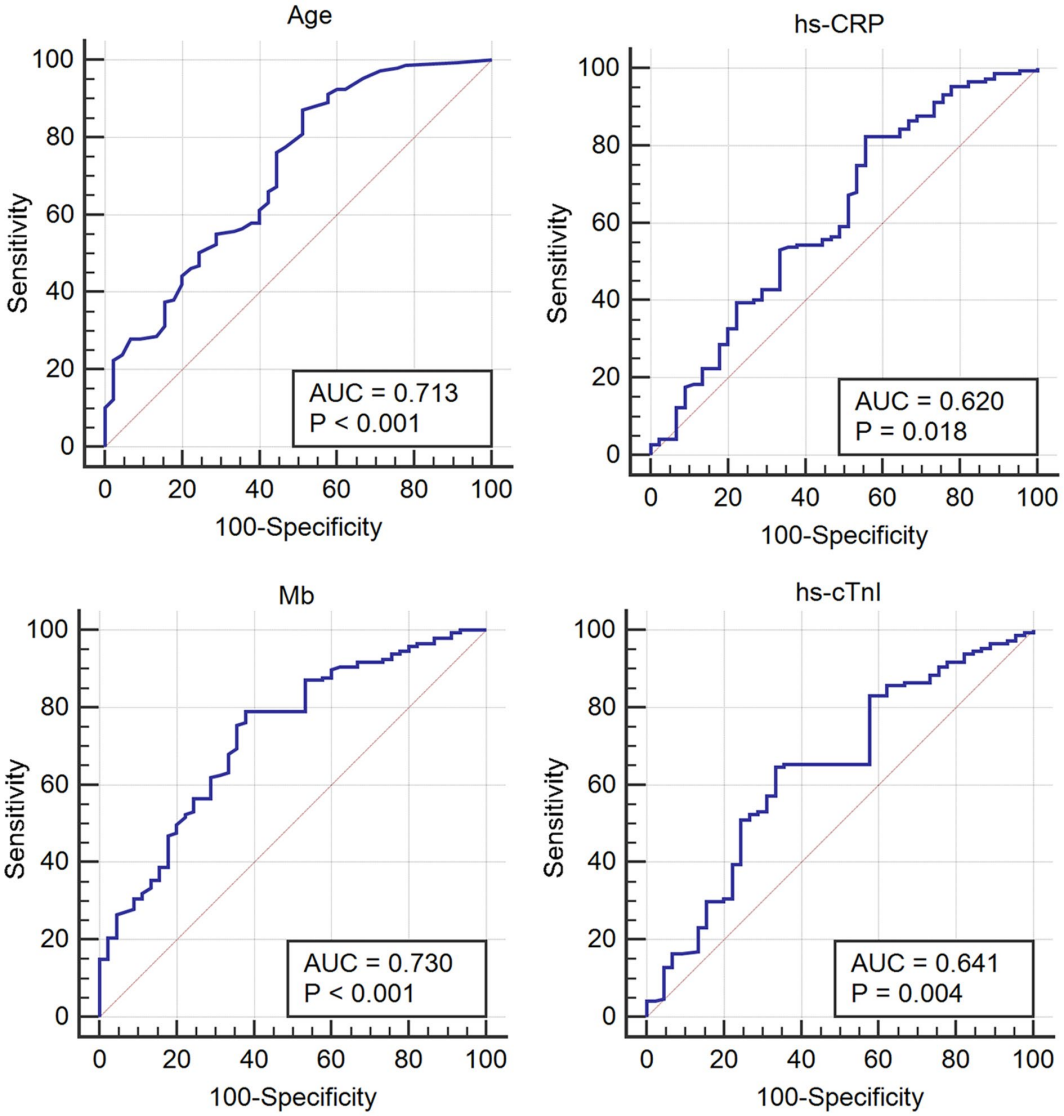
ROC curve of the quantified variables to obtain the optimal cut-off values for evaluating the prognosis of patients with Omicron COVID-19 complicated with arrhythmia.

The quantifiable variables, including age and hs-CRP, Mb and hs-cTnI levels, were analyzed by ROC curve to obtain their optimal cut-off values, for evaluating the prognosis of COVID-19-infected patients with arrhythmia in the future. The results demonstrated that the diagnostic cut-off values for age and hs-CRP, Mb and hs-cTnI levels were 69 years old, 10.19 mg/L, 63 U/L and 27.6 ng/L, respectively (Figure 2; Table 5).

**Table 5:** ROC curve analysis of the measurement variables that affect prognosis in arrhythmia patients infected with Omicron COVID-19.

Variables	AUC	Cut-off value	Sensitivity (%)	Specificity (%)
Age (years)	0.713	69	87.07	48.89
hs-CRP (mg/L)	0.620	10.19	82.31	44.44
Mb (U/L)	0.730	63.0	78.91	62.22
cTnI (ng/L)	0.641	27.6	64.63	66.67

## 4. Discussion

Increasing evidence has demonstrated that SARS-CoV-2 infection can lead to direct or indirect cardiac involvement and a series of cardiovascular complications including arrhythmia, which in severe cases can lead to heart failure, cardiogenic shock, cardiac arrest, syncope, and death (16). Omicron, one of the “worrying” variants identified by the World Health Organization, began to circulate in mainland China in December 2021, and became the most common SARS-CoV-2 variant (17). Arrhythmia is one of the most common complications after Omicron infection (18). By inspecting the clinical data of 192 patients with an Omicron infection complicated with arrhythmia, a preliminary analysis of the factors affecting these patients and their short-term prognosis was conducted in this study.

### 4.1. History of arrhythmia and fast heart rate

Arrhythmia is a symptom where the heart beats at an abnormal rate or rhythm, induced by the disorder of heart activity or conduction, and tachyarrhythmia is the main type of arrhythmia. Patients with a history of arrhythmia have a somewhat weaker beating rate and often have a faster heart rate than the average person when injured (19). After infection with SARS-CoV-2, patients are subjected to hypoxia and cytokine storms and appear to have sustained rapid heart rates to accelerate oxygen supply (20). Therefore, patients with a history of arrhythmia are more vulnerable to damage to the heart (even after the RNA test is negative) and find it difficult to recover quickly. Studies have demonstrated that COVID-19 can lead to arrhythmia and thus adverse disease outcomes, especially for infected patients with hereditary arrhythmia syndrome (21). Sacilotto et al. (22) believed that children with a history of arrhythmia, fever, electrolyte disturbance and drug effects caused by SARS-CoV-2 infection would most likely incur myocardial damage or aggravated hidden or well-controlled primary arrhythmia might occur. The results of the present study demonstrated that rapid heart rate during infection (≥100 times/min) was a risk factor for patients with concurrent arrhythmia, while a history of arrhythmia was a risk factor for poor prognosis of these patients, which was consistent with the results of existing studies (21, 22).

### 4.2. Myocardial function indexes

Myocardial function injury is recognized as one of the most common serious complications (23). In the early stages of the COVID-19 pandemic, several studies based on the autopsies of COVID-19 patients found large amounts of inflammatory infiltrates and epicardial edema in the heart tissues (24, 25). Current studies have found that the mechanism of myocardial injury caused by SARS-CoV-2 infection mainly includes the angiotensin converting enzyme 2 pathway, hypoxia and ischemic injury, inflammation and cytokine storm, and psychological stress (26–29). Myocardial enzyme assay is the main index to evaluate the status of myocardial injury. Sandoval et al. (30) found that elevated cardiac troponin I(cTnI) was common in COVID-19 patients and was positively correlated with the risk of adverse outcomes, such as arrhythmia and death. However, the cTnI level was generally lower than the diagnostic value of acute myocardial infarction. Henry et al. (31) conducted a meta-analysis of 24 biomarkers using data from 624 cases of children with COVID-19, and found that the pooled prevalence estimate of elevated CK-MB was 33% in mild cases. Moreover, other cardiac abnormalities caused by SARS-CoV-2 infection have also been reported another study (32). The results of the present study demonstrated that the elevation of CK-MB and hs-cTnI were risk factors for arrhythmia after SARS-CoV-2 infection, and that the elevation of Mb and hs-cTnI were risk factors for poor short-term prognosis of patients with arrhythmia. Therefore, it was suggested that myocardial indexes should be examined and cardiac protection should be performed immediately when symptoms of arrhythmia appear. It should be noted that a proportion (32 cases, 11%) of patients with heart disease, including chronic coronary atherosclerotic cardiopathy, in the present study may have a higher baseline myocardial index before infection than other infected patients. For such patients, the authors believe that slight elevation of myocardial injury markers has limited prognostic significance, and attention and reasonable response should be taken only in the case of significant elevation.

### 4.3. WBC and hs-CRP

Inflammatory cytokine storm is one of the biggest challenges faced by the body after SARS-CoV-2 infection, and the change of leukocytes is an intuitive indicator of immune status (33). In general, WBC levels will decrease after virus invasion and increase when combined with a bacterial infection. Therefore, it can be speculated that infection with bacteria and other pathogens, as evidenced by an increased WBC count, may be one of the causes of myocardial damage and thus arrhythmia in patients. CRP is also an inflammatory marker and it can activate complement and strengthen phagocytosis when the body is infected, to eliminate pathogenic microorganisms invading the body and consequently play an important protective role in the natural immune process. Therefore, within a certain range, elevated hs-CRP can play a role in the defense against infection. However, when the hs-CRP level is too high, it will cause immune damage, including myocardial damage, due to its involvement in inflammatory reactions (34). Studies have demonstrated that QT interval prolongation, a type of arrhythmia, is closely related to CRP levels in COVID-19 patients, and patients have higher CRP and WBC levels during hospitalization (35, 36). The results of the present study also demonstrated that an elevated WBC count is a risk factor for patients with arrhythmia, and that elevated hs-CRP is a protective factor for patients with arrhythmia after infection. However, hs-CRP is also a risk factor for poor prognosis, which is not completely consistent with existing studies. These inconsistencies may be related to the differences in test time points (during initial diagnosis or hospitalization). Combined with the results of the ROC curve analysis, the diagnostic criteria for CRP can be further refined, for example: When the CRP level is 8.49–10.19 mg/L, the risk of patients with arrhythmias is reduced; when the CRP level is >10.19 mg/L, the risk of poor prognosis is increased.

### 4.4. Other factors

The results of the present study demonstrated that obesity (BMI ≥24 kg/m^2^) and moderate disease severity were risk factors for arrhythmia in patients infected with Omicron, and advanced age (≥65 years old) and moderate disease severity were risk factors for poor prognosis. Advanced age and obesity have previously been confirmed to be risk factors for arrhythmia (37), and studies have also confirmed that advanced age and severity of the disease are important factors influencing the poor prognosis of patients with COVID-19 (38, 39). According to the Diagnosis and Treatment Protocol for Novel Coronavirus Infection (Trial 10th Edition) published by the National Health Commission of China (15), the criteria for moderate disease severity is that imaging features of virus-infected pneumonia can be observed, therefore the risk of myocardial damage and arrhythmia for these patients is higher. In addition, in this, it was also found that vaccination was one of the protective factors for a good outcome in patients with arrhythmia. According to an existing study, vaccination is a protective factor for patients with consistent progression to severe or critical disease after infection (40), which may also be the reason for the low risk of poor prognosis in patients with arrhythmia. It is worth noting that only chronic comorbidities that are more common in the Chinese population were included in the present study, while other diseases such as chronic kidney disease, chronic gastritis and chronic anemia (which have a low incidence in the population or no studies have shown that they are related to the occurrence of arrhythmia) were not included. These patients with these specific medical histories would have been excluded from the present study, but patients were not actively surveyed for every chronic disease. Therefore, it cannot be ruled out that certain patients may have a special medical history. However, we believe that such patients are a small proportion of the patients included and not enough to affect the conclusion of the present study.

Additionally, the limitation of the present study is that the subtype of Omicron virus was not further detected and analyzed. The reason for this is that cases were collected during the first round of explosive infection after the relaxation of epidemic prevention policies in mainland China, and too many infected patients were admitted meaning that medical staff had to devote all their energy to receiving and treating patients. In addition, the treatment and examination process was accelerated as much as possible. Therefore, the identification of virus subtypes in patient samples was not conducted. In addition, the present study was limited by a short follow-up time, which was 6–8 weeks for all patients. Later, we followed up the recovery of most patients with a poor prognosis and found that some patients improved in 10–12 months. A small number of patients found it difficult to fully recover and, compared with before infection, their physical fitness decreased significantly, especially in strenuous exercise and high-load work, where there were more notable cardiac discomfort symptoms. However, whether these patients need more time (>6 months) for recovery or whether these patients will develop “long COVID” needs to be followed up to have a clear conclusion. In future studies, we will pay further attention to the differences in symptom manifestations among those infected with different Omicron subtypes, continue to expand the sample size and extend the follow-up time to obtain more accurate conclusions.

## 5. Conclusion

The results of the present study demonstrated that the factors affecting arrhythmia in patients with Omicron COVID-19 include obesity (BMI ≥24 kg/m^2^), fast heart rate (≥100 times/min), moderate disease severity, and WBC, CK-MB and hs-cTnI levels. The factors affecting prognosis of Omicron COVID-19-infected patients with arrhythmia include advanced age (≥65 years old), history of arrhythmia, moderate disease severity, and hs-CRP, Mb and cTnI levels, which can be used to evaluate and prevent arrhythmia complications in patients with Omicron COVID-19 in the future.

## Data availability statement

The raw data supporting the conclusions of this article will be made available by the authors, without undue reservation.

## Ethics statement

The studies involving human participants were reviewed and approved by Ethics Committee of the Henan Provincial People’s Hospital. The patients/participants provided their written informed consent to participate in this study.

## Author contributions

LY: conception and design of study, acquisition of clinical data, drafting of article. JW and XF: acquisition of clinical data. JL and LZ: acquisition of laboratory data. JH and XL: analysis of data. YS: language translation. FZ and XX: review, proofread and revision. XC and HY: conception and design of study, critical revision. All authors contributed to the article and approved the submitted version.

## Funding

This research was funded by the Henan Province Young and middle-aged Health Innovation Outstanding young talents training project (No.YXKC2021051), Henan Province high-level talent internationalization training funding project [Yuke(2022)-3], and Henan Province medical Science and technology project joint construction (No. LHGJ20210110).

## Conflict of interest

The authors declare that the research was conducted in the absence of any commercial or financial relationships that could be construed as a potential conflict of interest.

## Publisher’s note

All claims expressed in this article are solely those of the authors and do not necessarily represent those of their affiliated organizations, or those of the publisher, the editors and the reviewers. Any product that may be evaluated in this article, or claim that may be made by its manufacturer, is not guaranteed or endorsed by the publisher.
